# Association between physical and mental health in school-going adolescents: Sex-specific patterns of maladjustment in formative years of life

**DOI:** 10.1371/journal.pmen.0000708

**Published:** 2026-09-11

**Authors:** Varsha Singh, Harish Chaudhury, Mahim Sagar

**Affiliations:** 1 Department of Humanities and Social Sciences, Indian Institute of Technology (IIT) Delhi, Hauz Khas, New Delhi, India; 2 Department of Management Studies, Vishwakarma Bhavan, Saheed Jeet Singh Marg, Indian Institute of Technology (IIT) Delhi, Hauz Khas, New Delhi, India; 3 Department of Management Studies, Vishwakarma Bhavan, Saheed Jeet Singh Marg, Indian Institute of Technology (IIT) Delhi, Hauz Khas, New Delhi, India; PLOS: Public Library of Science, UNITED KINGDOM OF GREAT BRITAIN AND NORTHERN IRELAND

## Abstract

Adolescence is a turbulent period of rapid biopsychosocial changes that help transition to adulthood. Adolescence maladjustment shows sex-specific vulnerability such that internalizing pathologies are linked with depression-anxiety in females, whereas externalizing problems linked are with substance abuse and conduct disorders in males. Developing countries with patriarchal societies produce early gender-inequity that might impact physical health, family, and social environments of adolescents impacting adulthood. The link between sex-differences in physical health, social support (school & family) and adolescent problems in non-western context is unclear. Hence, adolescents from 19 schools (N = 5121, male = 48%, female = 39%, missing = 13%) filled Indian Adolescent Health Questionnaire to measure physical health, family, and social environment, Strengths and Difficulties Questionnaire was used for adjustment problems (internalizing-externalizing). Males reported better physical health and nutrition whereas females had better attitude towards safety, substance use, and academics. Physical health, family, school and social environment were correlated (*p* = .00), t-test showed sex-differences in all subdomains exce*p*t hygiene and family environment. For adjustment problems, 22.8% of school students experienced borderline problems and 16% were categorized highly problematic, male students had higher maladjustment problems. Contrary to the West, internalizing problems showed no sex-differences, but male students’ internalizing problems were due to peers. Partial correlation controlling sex and class showed that family support negatively correlated with emotion problems (r = - .05, p < .05), hygiene attitude positively correlated with hyperactivity problems (r = .033, p < .05). School intervention involving families might shield adolescents from mental health problems, targeting sex-specific risks such as nutrition and physical activity for females or drugs and safety for males might improve health. Using standard health measures might allow follow-ups for comparing health gender-gap across the developmental phases of individuals accounting for urban-rural divide accentuated in developing countries and diverse population.

## Introduction

Adolescence is a critical phase that lies between childhood and youth, it is characterized by rapid pace of physical and cognitive development. The developmental changes occurring in adolescence are shaped by biological and social factors (e.g., sex hormones and gender-specific roles and norms) and cognitive-affective changes such as interests, motivation, aspirations some of which might be learned from peers and family. Due to such rapid changes, adolescence requires considerable adjustments, increasingly, adolescence maladjusted is considered as a precursor to mental health problems in adulthood [1]. According to the parents and the teachers, adolescent males show high maladjustment [2]. However, sex-specific patterns have been observed, such as adolescent females are more likely to internalize their thoughts and emotions putting them at risk for affective disorders such as depression-anxiety), whereas adolescent males are more likely to externalize, and act out aggression putting them at greater risk for addiction and conduct disorder [3]. How do adolescents perceive their social, academic, family environment and whether there are sex-differences in how they experience and think about their physical health, school and academics, social peers and family environment could be related to their ability to adjust with adolescence as a phase. Attitude towards physical appearances, health, safety and the social environment (school, friends, and family) might differ between the sexes in non-western-countries where the macro environment is characterized by low gender equity, prevalence of patriarchy, poverty, poor education level, low literacy rate, with sex-differences intensified by differential parental investment due to intensified competition for resources. Adolescent maladjustment might reflect biological and social factors that contribute to sex-specific predisposition to problems, and identifying factors associated with adolescent maladjustment might enable timely detection of mental health problems [4]. Patriarchal societies show high parental investment in male child, experiencing inequity in parental investment in early life might contribute to gender-gap in health and education outcomes in adolescence. For example, parents send male child to expensive, distant school, investing in commute; higher parental investment will increase expectations of academic achievement for male student. On the other hand, female child might be enrolled in inexpensive local school which reduced travel time and distance travelled, saves commute expenses, ensures girl-safety, increasing the expectations of her availability for caring and household work, reducing expectations of academic achievement. The developed resource-enriched countries (e.g., availability of schools, quality of local schools, public transport and connectivity) might offer sense of physical safety, hygiene, socioeconomic and academic conditions more be conducive for mitigating adolescence maladjustment problems. Although sex and gender are different constructs but aligned with the biopsychosocial model, the two are highly inter-linked such that sex-differences might shape gender roles which can further amplify sex-specific vulnerabilities more prominently in male-centric patriarchal societies of developing countries. Therefore, we restrict the use of ‘gender’ to instances when gender roles and social expectations are exclusively discussed and continue to use ‘sex’ for uniformity, acknowledging that the two terms are distinct but deeply integrated. Despite offering a sharper contrast to sex-differences observed in western developed countries, sex-differences in adolescent mental health problems within the biopsychosocial framework is rarely examined in Asian countries [5]. It is unclear if school-going adolescents of an Indian capital city experience physical health, family and school environment in a sex-specific manner and show differences, and if these differences are related to sex-specific vulnerabilities in the form of internalizing-externalizing problems experienced by adolescents in developed countries.

The Western and Asian countries differ in literacy, employment, standard of living, health, nutrition and safety but maintain a consistent gender-gap showing a male-advantage in education, health, employment. It is unclear if these macrolevel environments translate into sex-specific patterns of physical and mental health and percolate in schools and community in ways that impact school-going adolescents shaping their formative years. Longitudinal datasets and follow-up studies pose a methodological challenge for researchers in developing countries due to which, sex-specific patterns of adolescent maladjustment problems and its trajectory in adulthood remains unclear in counties like India. Despite cultural and socioeconomic differences, sex-specific pattern of affective problems prevails in India (i.e., high depression – anxiety comorbidity in women) [6]. Although studies in the Western countries suggests that male adolescents have higher maladjustment in adolescence [2,7], patriarchal system that favors males might mitigate the extent of adolescence maladjustment experienced by male adolescent students. Reports of male maladjustment in Asian countries are inconsistent such as in Japan [8], and in China where maladjustment and internalizing problems showed no sex differences (age 6–15 years) [9]. A recent metanalytic review with studies from multiple Indian cities showed higher prevalence of depression in females [10]. Reports of maladjustment in Indian males are unclear, some studies reported no sex differences in maladjustment (13–17 years) [11,12] while others report that female adolescents have higher maladjustment [13–15]). Internalizing – externalizing problems show a trend similar to that observed in western, developed countries where adolescent females report higher emotion problems and adolescent males report greater conduct problems in India [11,14,16]. Sex-specific vulnerabilities reported in Indian adolescents are not exactly similar to those of western population, for instance, conduct problems are not restricted to male adolescents as observed in the western sample (e.g., [12,13,15]). Sex-differences in adolescent maladjustment in the form of high externalizing problems in adolescent males and high internalizing problems in adolescent females remain poorly understood in Asian country such as India. Therefore, the study aimed to examine sex-differences in maladjustment problems in school-going adolescents to test if sex-specific trends observed in the non-western countries occur for the school-going adolescents from an Indian city.

Developing countries with patriarchal societies where the population has strong ‘son-preference’, a girl-child’s experiences of the physical, socioeconomic, cultural and academic environment differs from that of a male child, the most direct impact might be inequity in resources that determine physical, social and cognitive development of the child, especially parental investment and access to resources. For instance, in terms of home and family influences, girl infants receive less nutrition in early life [17]. Indirect impact might be through gender socialization which enforces gender roles through norms that train and reward a girl child for behavior that reflects higher levels of self-sacrifice, respect, obedience, rule-compliance compared to a same-age male child, creating conditions that are conducive for internalizing behavior in a girl child from an early age (i.e., learning to control/suppress and direct negative emotions inwards rather than expressing them outwards). On the other hand, gender norms for male child might train and reward him for independence, heroic machoism, aggression and risk taking reinforced via higher participation in sports and social interactions, thereby creating conditions conducive for externalizing behavior (i.e., directing negative emotions outwards rather than holding or directing them inwards). Adolescent males and females might experience school environment differently impacting their time and attentional investment in academics and influencing their cognitive development. For instance, compared to school-going boys, adolescent girls show lower cognitive performance in school [18] and indulge less in physical activity [19]. Although there was no difference observed in academic competency, teachers rate female students higher in social competency, and it is the school’s social environment that pressurizes male adolescent students to gain higher academic competency and female adolescents towards excelling in social competency [20]. Hence aligned with the biopsychosocial model, developing countries with poor gender-equity create conditions conducive for sex-specific vulnerabilities in the formative years of adolescence when young child transits towards adulthood. It is unclear whether there are gender-differences in how adolescents perceive their physical health, safety, school academics, and family environment how whether the way they adjust to changes brought on by adolescence shows sex-specific patterns of maladjustment similar to what is observed in the western developed countries. The interrelationship between measures of physical, social, social and family environment, and with adolescent maladjustment problems in school-going Indian adolescents remains unexamined. Using a large sample representative of socio-economic variability (private – public schools from four zones), we examined the association between measures of physical health, social, school, family environment, tested sex-differences in health measure in physical, social, and family environment and, examined sex-specific maladjustment problems to test if trends observed in western countries hold true for school-going adolescents in an Indian city (i.e., prominent externalizing problems in males, and internalizing problems in females with high overall maladjustment in males). We hypothesized that there will be sex differences in perceived physical health and social context of the family and school as assessed by IAHQ, and maladjustment problems experienced by the adolescents as assessed by SDQ, we expected that there will be an association between perception of physical health, social environment (school & family) and internalizing-externalizing problems in school-going adolescents.

## Methodology

### Sample

The study employed a large sample from a capital city in India, drawing from nineteen schools representing both public (subsidized education) and private schools and collecting responses from students of grade IX to grade XII. Private and public schools from four zones (East, West, North, South) within the national capital region were approached for the study with permission from the regional regulatory authority taken for school participation. School principals facilitated health survey among school-going adolescents as a part of Health Awareness Campaign. The survey questionnaire was selected by VS and survey was carried out by the co-authors HC and coordinated by MS. Eleven private schools and eight of the fourteen government schools responded favorably and participated in the survey. Students of grades IX, X, XI, and XII filled the survey in a group setting. Total of 5121 adolescents participated in the study (male = 48%, female = 39%, missing sex information = 13%; grades IX & X = 66%, grades XI & XII = 34%). The study was carried out in the academic session spanning the month of March and April 2019 under the supervision of the author HC, the research team visited the schools and started the data collection from 11/03/2019 – 16/04/2019 (DD/MM/YYYY).

### Measures

a. Indian Adolescent Health Questionnaire (IAHQ: [21]): The questionnaire developed on school-going adolescents in India to assess health needs in adolescents in broad domains such as demographic information, physical health and activity (10 questions), nutrition (13), hygiene (5), medical & HIV (10), substance use (13), violence and injury (11), school academics (7) and family environment (14). The questionnaire covered multiple domains, but questions related to medical, HIV section and violence and injury section were deemed sensitive by the schools and excluded (pl see S1 Appendix). The author’s permission was obtained to modify the questionnaire and its scoring.
b. Strengths and difficulties questionnaire (SDQ; [22]): The questionnaire assesses mental health challenges in four domains (emotion, conduct, peer, hyperactivity) and prosocial behavior; each domain is assessed using five items to be rated to indicate the extent to which they are true using a 3-point rating scale (i.e., not true, somewhat true, certainly true), there are 25 statements that are rated and scored to reflect domains of maladjustment problems experienced by the participant with higher scores indicative of more maladjustment problems

### Design, variables, analyses

The IAHQ had statements and questions that were administered and these varied in response format (e.g., likert scale of 3-, 4-, 5-, and 7-point rating scales) including 16 questions with binary yes/no responses that were excluded (see [21] appendix for original questionnaire). Permission from authors of IAHQ was obtained for the modifications, and responses from 64 questions of IAHQ questionnaires were analyzed in the present study. Questions were divided into seven domains: physical fitness (9 items), nutrition (13), hygiene (7), substance use (16), safety (6), family (9), and school environments (4). The raw data (participant’s responses) were converted into a uniform rating scale to ensure that the score range reflects pro-health attitude ranging from low/poor, moderate, and highly health-conducive response. Responses were converted into a rating scale 0 – 3 to reflect 3 as highly health-promoting attitude (please see S1 Appendix for question-wise conversion of responses). Because the domains had unequal number of questions, averages scores were obtained for each subdomain (i.e., sum of domain scores/number of questions in the domain). This method enabled comparability between domains and produced a composite measure of total scores of IAHQ where higher scores reflected a healthy attitude (please see data file for subdomain-wise scores, total scores, transformed scores, and average scores, allowing reproducibility of results). Correlation was used to determine the link between average score of subdomains of IAHQ and SDQ to understand how physical health, social environment and maladjustment problems might be interlinked. An independent sample t-test was used to examine sex differences in the subdomains of IAHQ and in SDQ to examine sex-specific patterns in physical and mental health respectively. Effect sizes were calculated from https://www.socscistatistics.com/effectsize/default3.aspx.

### Procedure

Schools in National Capital Region were approached to take part in a Health Awareness Campaign and study in school-going students. Approval was obtained from the regional commissioner (regulatory body) and school principals, informed consent was taken from students to enable their participation in the study. Parents were informed by the school, separate permission was not taken from the parents because schools maintained a rule that written parental approval is not taken if the activity is within the school premise, takes place within the school hours in a way that does not interfere with the regular school activity; present research was carried out as a part of the school’s efforts to improve health awareness in school children. The session was conducted by the research team during free period to minimize disruption of academic activity in school premises (e.g., assembly hall). On a designated day, the research assistants distributed the survey (booklet) to students seated in a large hall; the survey was administered one grade/division at one time and was to be filled in a single session lasting a school period (45 mins). Students gave verbal consent and participated in the survey, they were assured of anonymity and confidentiality and were encouraged to answer the survey without consulting or discussing among themselves.

After all survey responses were collected from all the classes, the team carried out an interactive engagement session where students were shown video developed to emphasize the importance of mental and physical health. Research assistants thanked the students for their time; all responses were entered in a data sheet and coded for analysis. Access to raw data was restricted to the investigators, a research coordinator, and two research assistants who entered the data.

## Results

Descriptive statistics allowed comparison of different domains of Indian Adolescent Health Questionnaire (IAHQ). For instance, attitude towards nutrition was poor whereas that towards substance use was pro-health among the adolescent students (Table 1). The domains of physical health, academic and social environment of school, and family were interlinked, results of correlation suggested that the IAHQ sub-domains were strongly correlated, physical activity positively correlated with nutrition (r = .42) and attitude towards hygiene (r = .32). Attitude towards substance use avoidance positively correlated with attitude towards safety (r = .27). Family environment and academics showed a positive correlation (r = .57) (Table 2).

**Table 1:** Descriptive statistics of Indian Adolescent Health Questionnaire (IAHQ) domain-wise average response.

IAHQ domains (no. of items)	N	Range	Mean (SD)
Physical activity (9)	4351	0 – 3.00	2.12 (.44)
Nutrition (13)	4352	0 – 3.00	1.99 (.44)
Hygiene (7)	4352	0 – 3.00	2.50 (.47)
Substance use (16)	4254	0 – 3.00	2.62 (.58)
Safety (6)	4352	0 – 2.83	2.34 (.37)
Family involvement (9)	4203	0 – 3.00	2.08 (.56)
Academic importance (4)	4352	0 – 3.00	2.27 (.67)
Total IAHQ (64)	4113	0 − 2.83	2.29 (.29)

Note: High scores indicate more pro-health/health-promoting attitude.

**Table 2:** Correlation between domains of Indian Adolescent Health Questionnaire (IAHQ: *N* = 4113, *df* = 4109, all correlation significant at *p* = .000).

	Phy. Activity	Nutrition	Hygiene	Substance	Safety	Family	Academics
Phy. Activity	1						
Nutrition	0.42	1					
Hygiene	0.32	0.43	1				
Substance	0.14	0.14	0.30	1			
Safety	0.14	0.08	0.24	0.27	1		
Family	0.20	0.20	0.21	0.09	0.22	1	
Academics	0.14	0.13	0.15	0.09	0.20	0.57	1

Although the responses were not normally distributed, usage of an independent sample t-test was justified based on the large sample size [23]. Results of t-test suggested that there was a significant difference such that adolescent males viewed physical fitness and nutrition more positively than adolescent females. On the other hand, adolescent females expressed awareness and healthier attitude towards substance usage, safety, giving importance to academics compared to adolescent males (Table 3). Adolescent male and female students did not differ in their attitude towards hygiene and the family environment, indicating that both valued these aspects equally. The total health risk scores showed no sex differences in school-going adolescents, it suggests that sex differences in health risk are domain-specific, the sex-specific patterns emerged on examining separate aspects of health risks such as physical health, social environment, safety, school and academic.

**Table 3:** Independent sample t test and effect size for sex-differences across Indian Adolescent Health Questionnaire (IAHQ) domains.

	Males			Females					
	N	Mean	SD	N	Mean	SD	*t*	*p*	Cohen’s *d*
Phy. Activity	2379	2.16	0.44	1972	2.08	0.44	6.15	.000	0.18
Nutrition	2379	2.01	0.43	1973	1.95	0.44	4.36	.000	0.14
Hygiene	2379	2.50	0.46	1973	2.49	0.48	0.97	.333	0.02
Substance	2323	2.59	0.61	1931	2.65	0.53	-3.50	.000	0.13
Safety	2379	2.33	0.38	1973	2.36	0.36	-3.16	.002	0.08
Family	2296	2.08	0.56	1907	2.07	0.57	0.69	.492	0.02
Academics	2379	2.24	0.68	1973	2.31	0.66	-3.41	.001	0.10
IAHQ risk	2243	2.29	0.29	1870	2.28	0.30	0.25	.802	0.03

Note: For Nutrition, Hygiene, and Substance use domains, Levene’s test was significant (*p* < .05) therefore t values for equality of variance are not assumed for these domains. High scores reflects attitude conducive or desirable in promoting health.

Next, results from the Strength and difficulties questionnaire (SDQ) questionnaire using cut-offs that are commonly used in mental health studies showed that 61% of the school-adolescents sampled in the study might be well-adjusted but 22% were experiencing borderline adjustment problems, and 16% were experiencing severe maladjustment classified as problematic category. In other words, 39% of the school-going adolescents surveyed were in the borderline or problematic category in terms of the maladjustment problems. The largest proportion of those classified in the problematic category could be experiencing conduct problems (26%), maximum proportion of those categorized as borderline category were those experiencing peer problems. Maximum proportion of the well-adjusted participants were least likely to experience hyperactive-inattention problems, prosociality and better regulation of emotion problems might be characteristics of well-adjusted adolescents (Table 4).

**Table 4:** Descriptive statistics of Strengths and difficulties questionnaire (SDQ: UK cut-offs).

Domains (cut-off for % of normal, borderline, problematic)	N	Mean (SD)	Normal %	Borderline %	Problematic %
Emotion problems (0–5,6, 7–10)	3276	3.72 (2.20)	79.0	09.5	11.5
Conduct problems (0–3,4, 5–10)	3671	3.35 (1.90)	56.4	17.8	25.8
Hyperactive-inattention (0–5,6,7–10)	3696	3.79 (1.89)	81.1	10.9	08.0
Peer problems (0–3,4–5, 6–10)	3511	3.24 (1.89)	57.9	29.0	13.0
Prosocial behavior (6–10, 5, 0–4)	3743	7.25 (2.06)	80.1	09.0	11.0
Total SDQ (0–15, 16–19, 20–40)	3083	14.01 (5.32)	61.2	22.8	16.0

Note: High scores reflect higher mental health challenge for all the domains except in prosocial behavior, wherein higher scores reflect high prosocial behavior.

Sex-specific patterns were observed in Strengths and difficulties questionnaire (SDQ) categories, firstly, adolescent males experienced maladjustment more severe than adolescent females as the combined percentage of borderline and problematic category for adolescent male student is higher (29.6%). Adolescent female students experiencing borderline and problematic level maladjustment were more likely to experience problems in the domain of emotion problems, whereas adolescent male students who were categorized as borderline and problematic in terms of maladjustment experienced conduct and peer related problems (Table 5). Results of independent sample t test suggested the most prominent sex differences were observed in externalizing problems, whereas internalizing problems showed no sex-differences (Table 6). Counter-intuitively, there were no significant difference between adolescent male and female students when it came to internalizing problems, but nature of problem and source of distress differed between the sexes - female students experienced more emotion problems whereas males experienced more peer problems. It is possible that when the two domains of emotion and peer related problems were are combined (as is customary to get a domain score of internalizing – externalizing problems), the differences canceled out and produced absence of sex-differences in internalizing behavior.

**Table 5:** Sex-specific Strengths and difficulties questionnaire (SDQ categories in domains).

SDQ subscale	Sex (missing %)	Normal %	Borderline %	Problematic %
Emotion problems	Male (14.8)	70.1	07.1	08.1
	Female (13.9)	64.8	09.4	12.0
Conduct problems	Male (15.5)	46.1	15.3	23.2
	Female (14.6)	50.2	14.9	20.3
Hyperactive-inatt.	Male (17.7)	65.0	09.9	07.3
	Female (21.2)	65.9	07.3	05.5
Peer problems	Male (15)	45.8	26.6	12.5
	Female (16.4)	52.6	21.9	09.2
Prosocial behavior	Male (14.2)	68.3	08.0	09.5
	Female (13.8)	69.6	07.3	09.3
Total SDQ	Male (28)	42.4	17.2	12.4
	Female (30.6)	44.6	14.9	10.0

**Table 6:** Independent sample t test and effect size for sex-difference across the domains of Strengths and difficulties questionnaire (SDQ).

	Males			Females					
SDQ subscale	N	Mean	SD	N	Mean	SD	*t*	*p*	Cohen’s *d*
Emotion	2027	3.46	2.15	1699	4.02	2.22	-7.68	.000	0.26
Conduct	2011	3.44	1.95	1685	3.24	1.84	3.18	.001	0.13
Hyperactive	1957	3.90	1.91	1554	3.66	1.87	3.60	.000	0.22
Peer prob.	2021	3.42	1.88	1650	3.01	1.88	6.62	.000	0.10
Prosocial	2042	7.11	2.05	1701	7.42	2.07	-4.49	.000	0.15
Internalizing	1923	6.86	3.25	1586	6.98	3.24	-1.10	.272	0.04
Externalizing	1856	7.34	3.12	1475	6.89	3.01	4.19	.000	0.15
Total SDQ	1713	14.19	5.39	1370	13.78	5.22	2.09	.036	0.07

Note: Levene’s test was significant (*p* < .05) for the conduct problem domain, and the t values for equality of variance are not assumed for this domain. High scores reflect higher mental health challenges for all the domains except in prosocial behavior, wherein higher scores reflect higher prosocial behavior.

To examine the link between internalizing – externalizing problems (SDQ) and how adolescent experience physical, social, academic, and family environment (IAHQ domains) we controlled for age (grade/class) and sex to identify pattern of maladjustment (internalizing – externalizing problems) and factors associated with it. Results indicate that emotion related problems were negatively correlated with healthy attitude towards safety at an unacceptable level of statistical significance (r = -.033, p < .10) and a supportive family environment was negatively correlated with emotion related problems (r = -.054, p < .05). Similarly, sensitivity to maintaining hygiene was positively correlated with experiencing peer problems (r = .033) at a 0.10 level of statistical significance; and with hyperactivity problems (r = .033, p < .05). When the domains of SDQ were combined to produce a single measure of maladjustment problems, family environment showed a negative correlation with internalizing problems indicating that supportive parental/family environment might be a protective factor against internalizing problems in school going adolescent students controlling for age and sex (Table 7).

**Table 7:** Partial correlation between the Indian Adolescent Health Questionnaire (IAHQ domains: physical health, family, school support) and Strengths and difficulties questionnaire (SDQ domains: emotion, conduct, hyperactivity, peer related problems) holding sex (male & female) and academic class (9 – 10^th^ as young & 11 – 12^th^ as older adolescents) as constant (N = 3000; df = 2996, p < .10*, < .05**, < .01***).

IAHQ domains	SDQ: Emotion	SDQ: Conduct	SDQ: Hyperactive	SDQ: Peer	SDQ: Internalizing	SDQ: Externalizing	Total SDQ
Phy. Act	.018	-.005	-.006	.027	.028	-.007	.013
Nutrition	.007	.012	-.005	.026	.020	.004	.013
Hygiene	-.014	.008	.037^**^	.031^*^	.009	.027	.016
Substance	-.006	-.024	-.012	.016	.005	-.022	-.008
Safety	-.033^*^	.022	.026	.020	-.011	.030	.004
Family	-.054^***^	-.022	.000	-.018	-.046^**^	-.014	-.027
Academics	.002	.009	.009	-.019	-.009	.011	.003
IAHQ	-.017	-.009	.007	.014	-.004	-.001	-.001

Note: Indian Adolescent Health Questionnaire (IAHQ) and its domains are scored such that higher value reflects pro-health, positive, favorable experience in that domain; Strengths and difficulties questionnaire (SDQ) and its domains are scores such that higher value reflects problem severity in that domain.

## Discussion

The study was aimed to understand physical and mental health of school-going adolescents to examine if sex-specific pattern of maladjustment to adolescence (i.e., internalizing problems in adolescent females and externalizing problems in adolescent males) are linked with how the sexes perceive their physical, social, and academic environment. Two surveys were used one was Indian Adolescent Health Questionnaire (IAHQ) that helped assess multiple domains of physical, social, and academic environment and another was Strengths and Difficulties Questionnaire (SDQ), a measure of maladjustment and internalizing – externalizing problems experienced by school-going adolescents, it is widely used across countries and is related to affective disorders risk in adulthood.

The results indicated that all subdomains of the IAHQ were positively correlated, indicating importance of all aspects of physical health, school academics, peers and family are important; having a positive attitude towards one might facilitate other domains as well. Next, we observed sex-differences in all domains except for hygiene and family environment where there were no significant sex differences. Adolescent female students had poor attitude towards nutrition and physical activity compared to adolescent male students. Behavioral change and intervention theories identify two domains, nutrition, and physical activity, as the least responsive to planned change [24], hence these subdomains might need targeted intervention in school-going adolescents over and above the existing mandatory class of physical education offered in all schools. Low physical activity is a cause of concern because it is associated with poor mental health [25], specifically internalizing pathologies [26]. Adolescent females show low physical activity in developed countries [27], low nutrition and low physical activity may jointly contribute to internalizing problems in females however such causal link in case of Indian adolescent females is not clearly established as yet. Whether low nutrition and physical activity are due to gender stereotypes, and if female adolescents are disproportionately affected by absence of safe and affordable infrastructure that is conducive to promote physical activity (i.e., public parks, jogging tracks, sport complexes, gymnasiums), whether there is parental investment in sports that could promote physical activity. The possible bidirectional link between low physical activity and low nutrition also remains unclear but could be an important factor.

We further observed sex-differences in the subdomains of IAHQ questionnaire; for instance, the present sample showed a trend consistent with Western countries, that is, higher inclination towards substance use was observed in school-going adolescent male students. Substance use is associated with internalizing problems and depression in women [28], and it is now increasingly reported in females in countries such as Brazil [29]. There is a possibility that gender roles contribute to these sex differences; for instance, in case of adolescent females, substance use might be associated with socializing/rewarding virtuous behavior, rule-compliance, restraint, or economic factors such as financial resources (pocket money) to purchase drugs, access to drugs; for males, it might be associated with a misplaced sense of heroism, machoism, peer pressure, access to economic and social resources that enable easy access to drugs and alcohol. Further, males reported better family environment than females however, hygiene and family environment were the only domains of IAHQ that showed no sex differences. There was a strong association between family environment and the importance given to academics, indicating that a facilitative family environment might be conducive to academic learning. Others have also found that female adolescents value academic performance, whereas importance given to academic performance for male adolescents depends on the family’s socioeconomic status [30], potentially linked with career aspirations and socioeconomic mobility. For the IAHQ total scores, sex-difference was not significant suggesting that the differences are restricted to specific subdomains (e.g., nutrition & physical activity were favorable factors in male students, whereas attitude towards substance use, safety, and academics were favorable factors in female students). The results indicate that identifying specific domains for targeted interventions might improve health outcomes for both the sexes and potentially reduce the health disparities emerging in adulthood.

The SDQ showed that independent of sex, 22.8% school-going adolescents were categorized as borderline, and 16% were categorized as highly problematic in adolescence maladjustment problems. The authors of IAHQ reported SDQ scores where 9.5% of students from three private schools in India were categorized as highly problematic and higher maladjustment was observed in female students [25]. Compared to [25], the current study surveyed nineteen government and private schools from one state with a larger sample size, and hence align with the group in noting variability in reports of SDQ in Indian school-going adolescents. A metanalytic review reported 23.33% of school-going students in India experience mental health problems [31]. Others reported SDQ results from a school in northeastern region of India have observed 21.6% of school-going adolescents were at the highly problematic level, and 31.4% were at the borderline level and conduct problems (19.2%) and emotional problems (12.4%) were similar to ones observed in the present study, although sex-specific percentage were not reported [32]. Another study using SDQ in adolescent girls from southern state indicated very high conduct problems (45.8%), high emotional problems in school-going girls (22.5%) compared to non-school going girls (18.3%) and measures of health and quality of life influenced mental health problems of adolescent girls [33]. However, number of school-going adolescents with abnormal and borderline problems might be higher in the Northern part of the country indicative of within-country, state-level variation (e.g., [11,14,34]). High maladjustment problems might reflect the capital city’s characteristics namely high juvenile crime rates and gender-based violence compared to the other states [35]. Our results indicated that prevalence of conduct problems is 26%, and high proportion of students reported borderline severity in peer-related problems (29%). Large scale school-level interventions (e.g., Happiness Program, Delhi Government) might help reduce adjustment problems in school-going adolescents and planned behavioral training and interventions could be used to produce domain-specific changes, for example, timely screening for hyperactivity and attention deficit targeting adolescent male students, educational programs for nutrition and physical training for adolescent girl students, along educational programs targeting common problems associated with adolescence. Further, promoting prosocial behavior through curriculum or extracurricular activities (volunteering, gratitude programs) might promote prosocial behavior which in turn might help adolescents to cope with the changes taking place in their physical, social, and academic environment.

As expected, results of t-test align with the cultural and gender frame as adolescent female students showed fewer externalizing problems compared to adolescent male students. Externalizing behavior is detrimental for the individual and the society, however, recently there is an alternate view that considers externalizing behavior as a protective function against chronic stress, and there might be a need to re-interpret externalizing behavior in females for factors such as lack of agency, freedom that a trusting, encouraging, facilitative environment could provide [36]. The IAHQ results also suggested that there were significant sex differences in how family environment is perceived by adolescents. Even same parenting style produces sex-differences in adolescence maladjustment for instance, parental style of autonomy increases externalizing problems in males as compared to females, whereas parental regulation decreases aggression more in females compared to males [37]. Hence strict parenting might reduce externalizing in female adolescents, not in males. These results are of interest for sex differences in mental health because externalizing behavior increases with age in males, and gender gap in externalizing behavior is attributed to genetic and heredity factors in males, and to environment in females [38]. Counter to the expectations, SDQ results indicated that internalizing problems did not differ significantly between male and female adolescent students. Adolescent males reported experiencing internalizing problems as frequently as adolescent females– these results are culturally inconsistent because others have reported males experience low internalizing problems compared to females across cultures (e.g., [39]). An Indo-Dutch comparison found high internalizing behavior in young adolescents in India (10-year-olds) [40]. Others have also found anxiety, an internalizing behavior to be higher among males than female adolescents in India [41]. Male internalizing behavior might suggest that the ‘strong, stoic male’ gender stereotype where emotional problems are not experienced or as openly expressed as same-aged female adolescents might not hold true. Male internalizing problems were focused on peer group in the present sample, this insight is crucial because peers are a big influence in adolescents such that they influence eating habits [42] and initiation of tobacco consumption in male adolescents [43]. Interventions for in male school-going adolescents that target mitigating peer problems via training for understanding, expressing emotions, coping with peer-problems might improve physical and mental health.

Lastly, we examined the link between perceived physical health, social and family environment and adolescent maladjustment problems via measures of IAHQ and SDQ to understand how these might be interlinked independent of sex and age. The results from partial correlation showed that domains of physical health, family and school environment were correlated with SDQ maladjustment problems. Results are aligned with the authors of IAHQ, who surveyed three private schools from three Indian states (West Bengal, Haryana, Uttar Pradesh) and observed that better health attitude in IAHQ was associated with SDQ scores indicating better mental health, although the authors sex-specific nature of this link was not examined [25]. Safety and supportive family environment had a protective association potentially reducing emotion problems in school-going adolescents. On the other hand, attitude towards hygiene was associated with hyperactivity and peer-related problems. Most prominent association was between family environment serving as a protective factor against internalizing problems. Pandemic-related isolation might have increased the reliance on family. Family environment remains an important determinant of internalizing problems among school-going adolescents independent of age and sex and should be the focus of mental health interventions in school going adolescents.

## Conclusions and limitations

The results identified sex differences in specific domains of physical health, including social, family, and academic environments. Further, the study documented sex-specific patterns in health attitude inclusive of physical health, social, school/academic and family environment and found significant differences in how adolescent male and female students perceived these factors as they negotiate the changing demands of adolescence. Sex-specific patterns in mental health problems were observed in the form of internalizing-externalizing problems, results demonstrated some trends similar to those observed in the Western countries, that is adolescent male students experience higher maladjustment compared to female students, adolescent females experience low externalizing problems like other the western and non-western countries. However, in contrasts with observations in the Western countries where female adolescents show higher internalizing problems, we observed no difference between adolescent male and female students in terms of internalizing problems that they experienced implying that males at the stage of adolescents are as vulnerable as females in experiencing emotion turmoil and struggle with emotion regulation.

These results might be informative but are to be interpreted with limitations, self-reporting of responses especially for sensitive information of drug and alcohol usage might show effects of social desirability, increased in group-testing set up within the school premises. Because the analysis focused on sex-specific responses, missing data for response to identifying sex of the participant had to be excluded (13% of missing response for question), the authors of IAHQ also report significant missing responses [25]. We used ‘sex’ as biological sex of the participant and have not accounted for ‘gender’ which is a psychological variable, future studies should investigate in the order of increasing complexity ‘sex’, ‘gender’, and ‘gender-fluid’ categories to truly understand sex-differences in maladjustment problems. We did not use substitute/imputing methods for missing data because we believed it might indicate underlying attitude towards the domains, (i.e., what was being asked in the questionnaire) hence we reported missing data for each of the subdomain of the questionnaire. For instance, missing variables were higher in domains such as drug and alcohol, indicating lack of comfort to answer these questions. The responses were collected within the presence of research assistance, the sessions were held during school hours, and within the school premises, this might have increased social desirability. Although standardization was adopted for sessions, for instance all the instructions were printed and circulated to minimize the effect of research assistants’ interaction; however, we could not control factors including the time slots and days of the week that the schools allotted for the study. Lastly, all schools in Delhi/NCR region were invited to participate, however, those who responded favorably within the time frame of the study with a commitment to participate in the research, were included in the study, in absence of a prior sample size calculation, we foresee school-level self-selection bias might influence the findings. However, to our knowledge, the study has the largest sample size using the measures of SDQ and IAHQ and has reasonable representation from four zones (East, West, North, South), school type (public, private), gender/sex, diversifying demographics and mitigating some of the concerns about sampling and sample representativeness. Despite these limitations, the results provided crucial insights to sex-differences in physical health, and social environment, and how these might relate to mental health problems. The novelty of this work is that it is the only study that gives sex-specific descriptive statistics of IAHQ and SDQ responses allowing sex-specific insights to physical and mental health perceptions among school going adolescents in an Indian city. One improvement that the current results enable is conversion of IAHQ responses into a linear format that provides insights to health perception in adolescents. The measure is now widely used in India, but the data-related methods and steps are rarely discussed, such as unequal questions per subdomain, unequal response range, and variable response format (e.g., [32,44,45]). The score transformation carried out and reported in the present study might provide an easy and direct way to map variable responses set on a linear scale in ways that make it easy to interpret health perception in school-going adolescents. This methodological improvement might facilitate state-wise comparison within country, comparison with other countries. To enable comparison of sex-specific patterns observed in the Western and other non-western countries, or to identify protective factors such as family and the role that it plays in maladjustment problems, or for pre-post comparison of intervention effect. Results could be useful for planning sex-specific timely interventions to prevent and reduce the risk of mental health problems in schools. Adolescent male students from Asian countries might experience gender norms that differ from those prevalent in Western countries in ways that impact their help seeking behavior. These results might provide clear and specific goals for school-level mental health interventions.

## Supporting information

S1 Appendix Lists the section and question-wise response options that were provided to the participants, assigned values to the raw data of the responses received, transformed values to reflect desirable, pro-health responses (0 – 3). The transformed values were totaled and analyzed for each subsection.
(DOCX)
