# Supplementary material for: Association between physical and mental health in school-going adolescents: Sex-specific patterns of maladjustment in formative years of life
Source: PLOS Ment Health. 2026 Sep 11;3(9):e0000708. doi: 10.1371/journal.pmen.0000708 (PMC13567832; doi:10.1371/journal.pmen.0000708)
Supplement: Supplementary file 1 [file pmen.0000708.s001.docx]

S1 Appendix

Section and question-wise response options that were provided to the participants, assigned values to the raw data of the responses received, transformed values to reflect desirable, pro-health responses (0 – 3). The transformed values were totalled and analysed for each subsection.

**A] Physical health (1 – 9: 9 items)**

1. In general, how is your health?

| Responses | Missing Value | Poor | Fair | Good | Very good | Excellent |
| --- | --- | --- | --- | --- | --- | --- |
| Assigned Values | 0 | 1 | 2 | 3 | 4 | 5 |
| Transformed Values | 0 | 1 | 2 | 2 | 3 | 3 |

2. How do you describe your weight?

| Responses | Missing Data | Very underweight | Slightly underweight | Slightly overweight | Very overweight | About the right weight |
| --- | --- | --- | --- | --- | --- | --- |
| Assigned Values | 0 | 1 | 2 | 4 | 5 | 3 |
| Transformed Values | 0 | 1 | 1 | 2 | 2 | 3 |

3. Which of the following are you trying to do about your weight?

| Responses | Missing Data | Lose weight | Gain weight | I'm not trying to do anything about my weight | Stay the same |
| --- | --- | --- | --- | --- | --- |
| Assigned Values | 0 | 2 | 3 | 1 | 4 |
| Transformed Values | 0 | 1 | 1 | 2 | 3 |

4. How much sleep do you usually get each night during the school week? (state of health)

| Responses | Missing Data | Less than six hours a night | More than 10 hours a night | 6-7 hours a night | 8-9 hours a night |
| --- | --- | --- | --- | --- | --- |
| Assigned Values | 0 | 1 | 4 | 2 | 3 |
| Transformed Values | 0 | 1 | 2 | 3 | 3 |

5. During the last week, on how many days were you physically active for a total of atleast 30 minutes per day?

| Responses | No Record | Not aware/ Not keeping record | 0 days | 1 day | 2 days | 3 days | 4 days | 5 days | 6 days | 7 days |
| --- | --- | --- | --- | --- | --- | --- | --- | --- | --- | --- |
| Assigned Values | 1 | 3 | 2 | 4 | 5 | 6 | 7 | 8 | 9 | 10 |
| Transformed Values | 0 | 0 | 1 | 1 | 2 | 2 | 2 | 3 | 3 | 3 |

6. How important is it to you to feel like you are physically fit?

| Responses | No Record | Not too important | No Idea | Important | Somewhat Important | Very Important |
| --- | --- | --- | --- | --- | --- | --- |
| Assigned Values | 0 | 4 | 5 | 2 | 3 | 1 |
| Transformed Values | 0 | 1 | 1 | 2 | 2 | 3 |

7. Outside of school, how much time do you spend during a typical day sitting and watching television, playing on the computer, or doing other activities

| Responses | Not keeping a record | 5 to 6 hour per day | 7 to 8 hour per day | More than 8 hours per day | 3-4 hours per day | Less than 1 hour per day | 1 to 2 hour per day |
| --- | --- | --- | --- | --- | --- | --- | --- |
| Assigned Values | 1 | 5 | 6 | 7 | 4 | 2 | 3 |
| Transformed Values | 0 | 1 | 1 | 1 | 2 | 3 | 3 |

8. How many days in a week do you think you should be physically active for a total of at least 30 minute per day?

| Responses | I don't know | 0 day | 1 day | 2 days | 3 days | 4 days | 5 days | 6 days | 7 days |
| --- | --- | --- | --- | --- | --- | --- | --- | --- | --- |
| Assigned Values | 1 | 2 | 3 | 4 | 5 | 6 | 7 | 8 | 9 |
| Transformed Values | 0 | 1 | 1 | 2 | 2 | 2 | 3 | 3 | 3 |

9. Outside of school, how much time do you think you should spend during a typical day sitting and watching television, playing on the computer, or doing such activities

| Responses | I don't know | 5 to 6 hour per day | 7 to 8 hour per day | More than 8 hour per day | 3-4 hour per day | Less than 1 hour per day | 1 to 2 hour per day |
| --- | --- | --- | --- | --- | --- | --- | --- |
| Assigned Values | 1 | 5 | 6 | 7 | 4 | 2 | 3 |
| Transformed Values | 0 | 1 | 1 | 1 | 2 | 3 | 3 |

**B] Nutrition (10 – 22: 13 items)**

10. During the past week, how many times per day did you eat fruits such as apple, mango, banana, pineapple, papaya, jackfruit, guava or chickoo?

| Responses | Don't remember | I did not eat fruit in the past week | Less than one time per day | 1 time per day | 2 times per day | 3 times per day | 4 times per day | 5 or more time per day |
| --- | --- | --- | --- | --- | --- | --- | --- | --- |
| Assigned Values | 1 | 2 | 3 | 4 | 5 | 6 | 7 | 8 |
| Transformed Values | 0 | 1 | 1 | 2 | 2 | 3 | 3 | 3 |

11. During the past week, how many times per day did you eat vegetables, such as cauliflower, ladyfinger, pumpkin, brinjal, cabbage, spinach, peas, tomato, cucumber, beans?

| Responses | Don't remember / No record | I did not eat vegetable in the past week | Less than one time per day | 1 time per day | 2 times per day | 3 times per day | 4 times per day | 5 or more time per day |
| --- | --- | --- | --- | --- | --- | --- | --- | --- |
| Assigned Values | 1 | 2 | 3 | 4 | 5 | 6 | 7 | 8 |
| Transformed Values | 0 | 1 | 1 | 2 | 2 | 3 | 3 | 3 |

12. How often the vegetable you eat are cooked or fried in oil?

| Responses | No Record | Never | Rarely | Sometimes | Most of the time | Always |
| --- | --- | --- | --- | --- | --- | --- |
| Assigned Values | 6 | 1 | 2 | 3 | 4 | 5 |
| Transformed Values | 0 | 1 | 1 | 2 | 3 | 3 |

13. During the past week, how many times per day did you eat meat or fish, such as chicken, beef, mutton or shirmp?

| Responses | No Record | I did not eat meat or fish in past week | Less than one time per day | 1 time per day | 2 times per day | 3 times per day | 4 times per day | 5 or more time per day |
| --- | --- | --- | --- | --- | --- | --- | --- | --- |
| Assigned Values | 8 | 1 | 2 | 3 | 4 | 5 | 6 | 7 |
| Transformed Values | 0 | 1 | 2 | 2 | 3 | 3 | 3 | 3 |

14. During the past week, how many times per day did you drink carbonated soft drinks such as thums Up, Sprite, Seven Up Coke, Pepsi, Limka, Fanta?

| Responses | No Record | 3 times per day | 4 times per day | 5 or more time per day | 1 time per day | 2 times per day | I did not carbonated soft drinks during the past week | Less than one time per day |
| --- | --- | --- | --- | --- | --- | --- | --- | --- |
| Assigned Values | 8 | 5 | 6 | 7 | 3 | 4 | 1 | 2 |
| Transformed Values | 0 | 1 | 1 | 1 | 2 | 2 | 3 | 3 |

15. During the past week, how many times per day did you eat at a fast food restaurant or at places serving quick meal (eg Mc Donalds, KFC, Nirula's, Mongini's samosa, pizza, patty, berger etc?)

| Responses | No Record | 4 days | 5 days | 6 days | 7 days | 2 days | 3 days | 0 days | 1 days |
| --- | --- | --- | --- | --- | --- | --- | --- | --- | --- |
| Assigned Values | 9 | 5 | 6 | 7 | 8 | 3 | 4 | 1 | 2 |
| Transformed Values | 0 | 1 | 1 | 1 | 1 | 2 | 2 | 3 | 3 |

16. In your school have you been taught about the benefits of healthy eating, , including eating more fruits and vegetables?

| Responses | Missing | Unsure | No | Yes |
| --- | --- | --- | --- | --- |
| Assigned Values | 0 | 3 | 2 | 1 |
| Transformed Values | 0 | 1 | 2 | 3 |

17. How many times per day you think should eat fruits such as apple, mango, banana, pineapple, papaya, jackfruit, guava or chickoo?

| Responses | Missing data | I don’t know | Less than one time per day | 1 time per day | 2 times per day | 3 times per day | 4 times per day | 5 or more times per day |
| --- | --- | --- | --- | --- | --- | --- | --- | --- |
| Assigned Values | 0 | 1 | 2 | 3 | 4 | 5 | 6 | 7 |
| Transformed Values | 0 | 1 | 1 | 2 | 2 | 3 | 3 | 3 |

18. How many times per day think you should eat vegetables, such as cauliflower, ladyfinger, pumpkin, brinjal, cabbage, spinach, peas, tomato, cucumber, or beans?

| Responses | Missing data | I don’t know | Less than one time per day | 1 time per day | 2 times per day | 3 times per day | 4 times per day | 5 or more times per day |
| --- | --- | --- | --- | --- | --- | --- | --- | --- |
| Assigned Values | 0 | 1 | 2 | 3 | 4 | 5 | 6 | 7 |
| Transformed Values | 0 | 1 | 1 | 2 | 2 | 3 | 3 | 3 |

19. How often you think should eat the vegetable cooked or fried in oil?

| Responses | Don't know | Most of the time | Always | Sometimes | Rarely | Never |
| --- | --- | --- | --- | --- | --- | --- |
| Assigned Values | 6 | 4 | 5 | 3 | 2 | 1 |
| Transformed Values | 0 | 1 | 1 | 2 | 3 | 3 |

20. How many times per day a week you think you should eat meat or fish, such as chicken, mutton or shirmp?

| Responses | Don't know | 5 or more times per day | I am a vegetarian | Less than one time per day | 1 time per day | 2 times per day | 3 times per day | 4 times per day |
| --- | --- | --- | --- | --- | --- | --- | --- | --- |
| Assigned Values | 0 | 7 | 1 | 2 | 3 | 4 | 5 | 6 |
| Transformed Values | 0 | 0 | 1 | 2 | 2 | 3 | 3 | 3 |

21. How many times per day you week you think you should drink carbonated soft drinks such as thumbs Up, Sprite, Seven Up Coke, Pepsi, Limka, Fanta?

| Responses | Don't know | 4 times per day | 5 or more times per day | 2 times per day | 3 times per day | Less than one time per day | 1 time per day |
| --- | --- | --- | --- | --- | --- | --- | --- |
| Assigned Values | 7 | 5 | 6 | 3 | 4 | 1 | 2 |
| Transformed Values | 0 | 1 | 1 | 2 | 2 | 3 | 3 |

22. How many times per week you think you should eat at a fast food restaurant or at places serving quick meal (eg Mc Donalds, KFC, Nirula's, Mongini's samosa, pizza, patty, berger etc?)

| Responses | Don’t know | 4 days | 5 days | 6 days | 7 days | 2 days | 3 days | 0 days | 1 days |
| --- | --- | --- | --- | --- | --- | --- | --- | --- | --- |
| Assigned Values | 9 | 5 | 6 | 7 | 8 | 3 | 4 | 1 | 2 |
| Transformed Values | 0 | 1 | 1 | 1 | 1 | 2 | 2 | 3 | 3 |

**C] Hygiene (23 – 29: 7 items)**

23. During the past month how many times per day did you usually clean or brush your teeth?

| Responses | No record | I did not clean or brush my teeth during the past month | Less than one time per day | 1 time per day | 2 times per day | 3 times per day | 4 times per day | 5 or more times per day |
| --- | --- | --- | --- | --- | --- | --- | --- | --- |
| Assigned Values | 8 | 1 | 2 | 3 | 4 | 5 | 6 | 7 |
| Transformed Values | 0 | 1 | 2 | 2 | 3 | 3 | 3 | 3 |

24. During the past month how often did you wash your hands before eating?

| Responses | No record/Unsure | Never | Rarely | Sometimes | Most of the times | Always |
| --- | --- | --- | --- | --- | --- | --- |
| Assigned Values | 6 | 1 | 2 | 3 | 4 | 5 |
| Transformed Values | 0 | 1 | 1 | 2 | 2 | 3 |

25. During the past month how often did you wash your hands after using toilet or latrine?

| Responses | Unsure/No recall | Never | Rarely | Sometimes | Most of the times | Always |
| --- | --- | --- | --- | --- | --- | --- |
| Assigned Values | 6 | 1 | 2 | 3 | 4 | 5 |
| Transformed Values | 0 | 1 | 1 | 2 | 2 | 3 |

26. During the past month how often did you use soap while washing hands?

| Responses | Unsure/No recall | Never | Rarely | Sometimes | Most of the times | Always |
| --- | --- | --- | --- | --- | --- | --- |
| Assigned Values | 6 | 1 | 2 | 3 | 4 | 5 |
| Transformed Values | 0 | 1 | 1 | 2 | 2 | 3 |

27. How many times per day you think you should usually clean or brush your teeth?

| Responses | Don’t know | Less than one time per day | 1 time per day | 2 times per day | 3 times per day | 4 times per day |
| --- | --- | --- | --- | --- | --- | --- |
| Assigned Values | 6 | 1 | 2 | 3 | 4 | 5 |
| Transformed Values | 0 | 1 | 2 | 2 | 3 | 3 |

28. How often you think you should wash your hands before eating?

| Responses | Don’t know/Unsure | Never | Rarely | Sometimes | Most of the times | Always |
| --- | --- | --- | --- | --- | --- | --- |
| Assigned Values | 6 | 1 | 2 | 3 | 4 | 5 |
| Transformed Values | 0 | 1 | 2 | 2 | 3 | 3 |

29. How often you think you should wash your hands after using toilet or latrine?

| Responses | Unsure | Never | Rarely | Sometimes | Most of the times | Always |
| --- | --- | --- | --- | --- | --- | --- |
| Assigned Values | 6 | 1 | 2 | 3 | 4 | 5 |
| Transformed Values | 0 | 1 | 2 | 2 | 3 | 3 |

**D] Cigarette, Tobacco Use, Alcohol and Drugs (30 – 46: 16 items)**

30. Have you ever smoked cigarettes or chewed paan or guthka or paan masala?

| Responses | Unsure | Smoke regularly | Smoke occasionally | Tried a few times | Never |
| --- | --- | --- | --- | --- | --- |
| Assigned Values | 0 | 4 | 3 | 2 | 1 |
| Transformed Values | 0 | 1 | 2 | 3 | 4 |

31. During the past month, on how many days did you smoke cigarettes?

| Responses | Not sure | I do not smoke cigarettes | 10 or more days | Everyday | 3 to 9 days | 1 to 2 days |
| --- | --- | --- | --- | --- | --- | --- |
| Assigned Values | 0 | 1 | 4 | 5 | 3 | 2 |
| Transformed Values | 0 | 1 | 2 | 2 | 2 | 3 |

32. Have any one of your close friends ever tried smoking cigarettes or chewed ghthka or pan masala?

| Responses | Unsure | Yes | No |
| --- | --- | --- | --- |
| Assigned Values | 3 | 1 | 2 |
| Transformed Values | 0 | 1 | 2 |

33. Which of your parents or guardians smoke?

| Responses | Not sure | Both | Mother or female guardian | Father or male guardian | Neither |
| --- | --- | --- | --- | --- | --- |
| Assigned Values | 0 | 4 | 3 | 2 | 1 |
| Transformed Values | 0 | 1 | 2 | 3 | 4 |

34. Do you think smoking is harmful to your health?

| Responses | Not sure | No | Yes |
| --- | --- | --- | --- |
| Assigned Values | 0 | 2 | 1 |
| Transformed Values | 0 | 1 | 2 |

35. During the past month, have you ever seen any anti-smoking media messages (such as ads on television, radio, billboards, posters, newspapers magazines or movies)?

| Responses | Not sure | No | Yes |
| --- | --- | --- | --- |
| Assigned Values | 0 | 2 | 1 |
| Transformed Values | 0 | 1 | 2 |

36. How many days in month you think you will smoke cigarettes?

| Responses | Not sure | Everyday | 3 to 9 days | 10 or more days | 1 to 2 days | 0 day |
| --- | --- | --- | --- | --- | --- | --- |
| Assigned Values | 0 | 5 | 3 | 4 | 2 | 1 |
| Transformed Values | 0 | 1 | 2 | 2 | 3 | 4 |

37. Have you tried alcohol?

| Responses | Not sure | Yes | No |
| --- | --- | --- | --- |
| Assigned Values | 0 | 1 | 2 |
| Transformed Values | 0 | 1 | 2 |

38. In the past 6 months how many times have you tried alcohol?

| Responses | Not sure | Once a week | More than once a week | Once a month | More than once a month | A few times | Once | I have not tried alcohol |
| --- | --- | --- | --- | --- | --- | --- | --- | --- |
| Assigned Values | 0 | 6 | 7 | 4 | 5 | 3 | 2 | 1 |
| Transformed Values | 0 | 1 | 1 | 2 | 2 | 3 | 4 | 4 |

39. If you tried alcohol, where were you the first time you had a drink of alcohol?

| Responses | missing | At school | At a bar, pub, disco | In a restaurant | Out on the street or in a park | At home | At someone else's home | I have never tried alcohol |
| --- | --- | --- | --- | --- | --- | --- | --- | --- |
| Assigned Values | 0 | 5 | 3 | 4 | 7 | 2 | 6 | 1 |
| Transformed Values | 0 | 1 | 2 | 2 | 2 | 3 | 3 | 4 |

40. If you do drink alcohol, do you typically drink until you are intoxicated (drunk)?

| Responses | Dont know | No | I do not drink alcohol | Yes |
| --- | --- | --- | --- | --- |
| Assigned Values | 0 | 3 | 1 | 2 |
| Transformed Values | 0 | 1 | 2 | 3 |

41. Have any of your close friends ever tried beer, wine or other liquor (except for religious purpose)?

| Responses | Not sure | Unsure | Yes | No |
| --- | --- | --- | --- | --- |
| Assigned Values | 0 | 3 | 1 | 2 |
| Transformed Values | 0 | 0 | 1 | 2 |

42. In the next 6 months, how many times do you think you will try alcohol?

| Responses | not sure | I will not have alcohol even once | Once a week | More than once a week | Once a month | More than once a month | A few times | Once |
| --- | --- | --- | --- | --- | --- | --- | --- | --- |
| Assigned Values | 0 | 1 | 6 | 7 | 4 | 5 | 3 | 2 |
| Transformed Values | 0 | 1 | 1 | 1 | 2 | 2 | 3 | 4 |

43. Have any of your close friends ever used illegal drugs (example ganja, weed, pot, hash, charas, inhailing fluids, cracks, cocaine etc?)

| Responses | Unsure / not sure | Unsure | Yes | No |
| --- | --- | --- | --- | --- |
| Assigned Values | 0 | 3 | 1 | 2 |
| Transformed Values | 0 | 0 | 1 | 2 |

44. During the past year, how many times have you used illgal drugs (example ganja, weed, pot, hash, charas, inhailing fluids, cracks, cocaine etc?)

| Responses | unsure | 10 or more times | 3 to 9 times | 1 or 2 times | I have not illegal drugs in past year |
| --- | --- | --- | --- | --- | --- |
| Assigned Values | 0 | 4 | 3 | 2 | 1 |
| Transformed Values | 0 | 1 | 2 | 3 | 4 |

45. How many times in a month do you think on can use illgal drugs (example ganja, weed, pot, hash, charas, inhailing fluids, cracks, cocaine etc?)

| Responses | unsure | 10 or more times | 3 to 9 times | 1 or 2 times | 0 times |
| --- | --- | --- | --- | --- | --- |
| Assigned Values | 0 | 4 | 3 | 2 | 1 |
| Transformed Values | 0 | 1 | 2 | 3 | 4 |

**E] Safety (47 – 52: 6 items)**

46. Have you ever NOT GONE to school because you felt you would be unsafe either school on the way?

| Responses | not sure | Yes | No |
| --- | --- | --- | --- |
| Assigned Values | 0 | 1 | 2 |
| Transformed Values | 0 | 1 | 2 |

47. During the past year, how frequently have you experinced someone saying something internationally rude or insulting to you?

| Responses | not sure | Most of the times | Always | Rarely | Sometimes | Never |
| --- | --- | --- | --- | --- | --- | --- |
| Assigned Values | 0 | 4 | 5 | 2 | 3 | 1 |
| Transformed Values | 0 | 1 | 1 | 2 | 2 | 3 |

48. Have you ever seen a violent act take place at school or in your neighbourhood?

| Responses | not sure | Most of the times | Always | Rarely | Sometimes | Never |
| --- | --- | --- | --- | --- | --- | --- |
| Assigned Values | 0 | 4 | 5 | 2 | 3 | 1 |
| Transformed Values | 0 | 1 | 1 | 2 | 2 | 3 |

49. Do you feel safe at home?

| Responses | not sure | Never | Rarely | Sometimes | Most of the times | Always |
| --- | --- | --- | --- | --- | --- | --- |
| Assigned Values | 0 | 1 | 2 | 3 | 4 | 5 |
| Transformed Values | 0 | 1 | 2 | 2 | 3 | 3 |

50. Do you feel safe at School?

| Responses | not sure | Never | Rarely | Sometimes | Most of the times | Always |
| --- | --- | --- | --- | --- | --- | --- |
| Assigned Values | 0 | 1 | 2 | 3 | 4 | 5 |
| Transformed Values | 0 | 1 | 2 | 2 | 3 | 3 |

51. Do you feel safe when hanging out with friends?

| Responses | not sure | Never | Rarely | Sometimes | Most of the times | Always |
| --- | --- | --- | --- | --- | --- | --- |
| Assigned Values | 0 | 1 | 2 | 3 | 4 | 5 |
| Transformed Values | 0 | 1 | 2 | 2 | 3 | 3 |

**F] Family (53 – 61: 9 items)**

52. Do you live with both of your parents?

| Responses | missing data | Uncle | Aunt | Others | No, I live with my mother only | No, I live with my father only | Yes, I live with both parents |
| --- | --- | --- | --- | --- | --- | --- | --- |
| Assigned Values | 0 | 4 | 5 | 6 | 2 | 3 | 1 |
| Transformed Values | 0 | 1 | 1 | 1 | 2 | 2 | 3 |

53. Do you live with any other extended family members other than your perents and siblings?

| Responses | missing | Uncle | Aunt | Others | My mothers parents | My fathers parents | No, I don't live with my extended family |
| --- | --- | --- | --- | --- | --- | --- | --- |
| Assigned Values | 0 | 4 | 5 | 6 | 2 | 3 | 1 |
| Transformed Values | 0 | 1 | 1 | 1 | 2 | 2 | 3 |

54. During the past month, how often did your parents or guardians check to see if your homework was done?

| Responses | not sure | Never | Rarely | Sometimes | Most of the times | Always |
| --- | --- | --- | --- | --- | --- | --- |
| Assigned Values | 0 | 1 | 2 | 3 | 4 | 5 |
| Transformed Values | 0 | 1 | 1 | 2 | 3 | 3 |

55. During the past month, how often did your parents or guardians understand your problems?

| Responses | not sure | Never | Rarely | Sometimes | Most of the times | Always |
| --- | --- | --- | --- | --- | --- | --- |
| Assigned Values | 0 | 1 | 2 | 3 | 4 | 5 |
| Transformed Values | 0 | 1 | 1 | 2 | 3 | 3 |

56. During the past month, how often did your parents or guardians really know what you were doing in your free time?

| Responses | not sure | Never | Rarely | Sometimes | Most of the times | Always |
| --- | --- | --- | --- | --- | --- | --- |
| Assigned Values | 0 | 1 | 2 | 3 | 4 | 5 |
| Transformed Values | 0 | 1 | 1 | 2 | 3 | 3 |

57. How far did your father go in school?

| Responses | Unsure | Less than Primary school | Completed Primary school | Went to secondary school but did not graduate | Graduated from secondary school but did not go to college, university, or other training School | Went to college or university but did not graduate | Graduated from college or university | More education beyond college or university |
| --- | --- | --- | --- | --- | --- | --- | --- | --- |
| Assigned Values | 8 | 1 | 2 | 3 | 4 | 5 | 6 | 7 |
| Transformed Values | 0 | 1 | 1 | 2 | 2 | 2 | 3 | 3 |

58. How far did your mother go in school?

| Responses | Unsure | Less than Primary school | Completed Primary school | Went to secondary school but did not graduate | Graduated from secondary school but did not go to college, university, or other training School | Went to college or university but did not graduate | Graduated from college or university | More education beyond college or university |
| --- | --- | --- | --- | --- | --- | --- | --- | --- |
| Assigned Values | 8 | 1 | 2 | 3 | 4 | 5 | 6 | 7 |
| Transformed Values | 0 | 1 | 1 | 2 | 2 | 2 | 3 | 3 |

59. Is your father:

| Responses | Unsure | Stay at home father | Retired or disabled | Going to school, not working at job | Unemployed | Working at a job part time | Working at a job full time |
| --- | --- | --- | --- | --- | --- | --- | --- |
| Assigned Values | 7 | 3 | 4 | 5 | 6 | 2 | 1 |
| Transformed Values | 0 | 1 | 1 | 1 | 1 | 2 | 3 |

60. Is your Mother:

| Responses | Unsure | Stay at home mother | Retired or disabled | Going to school, not working at job | Unemployed | Working at a job part time | Working at a job full time |
| --- | --- | --- | --- | --- | --- | --- | --- |
| Assigned Values | 7 | 3 | 4 | 5 | 6 | 2 | 1 |
| Transformed Values | 0 | 1 | 1 | 1 | 1 | 2 | 3 |

**G] School environment (62 – 65: 4 items)**

61. On an average how are your marks in school?

| Responses | Missing | Very low | Low | Average | High | Very high |
| --- | --- | --- | --- | --- | --- | --- |
| Assigned Values |  | 1 | 2 | 3 | 4 | 5 |
| Transformed Values | 0 | 1 | 1 | 2 | 3 | 3 |

62. During the past month how often did you miss classes or school without permission?

| Responses | Missing response | 5 to 6 | 7 or more | 1 to 2 | 3 to 4 | 0 |
| --- | --- | --- | --- | --- | --- | --- |
| Assigned Values |  | 4 | 5 | 2 | 3 | 1 |
| Transformed Values | 0 | 1 | 1 | 2 | 2 | 3 |

63. How regularly do you something negative because you feel pressure from your peers?

| Responses | Missing response | Most of the times | Always | Sometimes | Never | Rarely |
| --- | --- | --- | --- | --- | --- | --- |
| Assigned Values |  | 4 | 5 | 3 | 1 | 2 |
| Transformed Values | 0 | 1 | 1 | 2 | 3 | 3 |

64. During the past month, how often were most of the students in your school kind and helpful?

| Responses | Missing response | Never | Rarely | Sometimes | Most of the times | Always |
| --- | --- | --- | --- | --- | --- | --- |
| Assigned Values |  | 1 | 2 | 3 | 4 | 5 |
| Transformed Values | 0 | 1 | 1 | 2 | 3 | 3 |
